# Correction: Multidrug resistance pattern of *Acinetobacter* species isolated from clinical specimens referred to the Ethiopian Public Health Institute: 2014 to 2018 trend anaylsis

**DOI:** 10.1371/journal.pone.0305646

**Published:** 2024-06-12

**Authors:** Zeleke Ayenew, Eyasu Tigabu, Elias Seyoum, Semira Ebrahim, Dawit Assefa, Estifanos Tsige

The third author’s name is spelled incorrectly. The correct name is: Elias Seyoum. The correct citation is: Ayenew Z, Tigabu E, Seyoum E, Ebrahim S, Assefa D, Tsige E (2021) Multidrug resistance pattern of Acinetobacter species isolated from clinical specimens referred to the Ethiopian Public Health Institute: 2014 to 2018 trend anaylsis. PLoS ONE 16(4): e0250896. https://doi.org/10.1371/journal.pone.0250896
