# Supplementary material for: Multidrug resistance pattern of Acinetobacter species isolated from clinical specimens referred to the Ethiopian Public Health Institute: 2014 to 2018 trend anaylsis
Source: PLoS One. 2021 Apr 29;16(4):e0250896. doi: 10.1371/journal.pone.0250896 (PMC8084144; doi:10.1371/journal.pone.0250896)
Supplement: Attachment — Submitted filename: Response to Reviwers.docx [file pone.0250896.s002.docx]

Journal Requirements:

When submitting your revision, we need you to address these additional requirements.

1. Please ensure that your manuscript meets PLOS ONE's style requirements, including those for file naming. The PLOS ONE style templates can be found at

<https://journals.plos.org/plosone/s/file?id=wjVg/PLOSOne_formatting_sample_main_body.pdf> and

<https://journals.plos.org/plosone/s/file?id=ba62/PLOSOne_formatting_sample_title_authors_affiliations.pdf>

**Response –Thank you editor, we have edited our manuscript based on the PLOS ONE's style requirements.**

2. Thank you for stating the following financial disclosure:

 [The funders had no role in study design, data collection and analysis, decision to publish, or preparation of the manuscript.].

At this time, please address the following queries:

a. Please clarify the sources of funding (financial or material support) for your study. List the grants or organizations that supported your study, including funding received from your institution.
b. State what role the funders took in the study. If the funders had no role in your study, please state: “The funders had no role in study design, data collection and analysis, decision to publish, or preparation of the manuscript.”
c. If any authors received a salary from any of your funders, please state which authors and which funders.
d. If you did not receive any funding for this study, please state: “The authors received no specific funding for this work.”

**Response – thank you editor! The authors received no specific funding for this work.” We have included this in updated cover letter.**

Please include your amended statements within your cover letter; we will change the online submission form on your behalf.

3. Your ethics statement should only appear in the Methods section of your manuscript. If your ethics statement is written in any section besides the Methods, please move it to the Methods section and delete it from any other section. Please ensure that your ethics statement is included in your manuscript, as the ethics statement entered into the online submission form will not be published alongside your manuscript.

**Response –thank you we included in the method section.**

4. Please ensure that you refer to Figure 4 in your text as, if accepted, production will need this reference to link the reader to the figure.

**Response –thank you we referred Figure 4 in the text with some modification.**

Reviewer #1: The manuscript describes the increase of resistance rates in Acinetobacter spp. strains, isolated from different types of samples, in Ethiopia. Although the authors presented interesting results, it is difficult for me to recommend this paper for publication in PLOS ONE in its present form.

1- The authors must include line numbers, as well as page numbers to help with the correction.

**Response – thank you reviewer for the suggestion to follow submission guidelines we included the line numbers and page number.**

2- The authors should revise the English and the style of the text. There are two types of fonts along the text.

**Response – thank you we revised the English and make the fonts uniform ‘Arial’.**

3- The authors should correct the nomenclature, in many cases, they have written Acinitobacter instead of Acinetobacter, and on several occasions, the word appears without italics.

**Response –yes we admitted this and we use ‘find and replace’ Acinitobacter with Acinetobacter throughout the text. Thank you for this important comment.**

4- The authors should homogenize the names of the antibiotics, whether with or without a capital letter, but all the same. The same applies for the percentages, between or not a parenthesis, but all the same in the same paragraph.

**Response – thank you, we make it small letter for and antibiotics and make it uniform for percentages, between or not a parenthesis, throughout the texts as highlighted yellow.**

5- The acronym HAI has two different explanations, one in the introduction and another one in the results.

**Response –thank you reviewer we make it uniform as the acronym HAI means to show hospital acquired infection.**
Introduction:
6- Line 7: The authors should explain the meaning of TU.

**Response –thank you -this is a genomic species of Acinetobacter which is given by taxonomy classification in addition to species level.**
7- The first time the name of a bacteria is mentioned should be written with its complete name. The subsequent times should be written in the reducing form of its name.

**Response – thank you for your comment also with exception at the beginning of a sentence, we wrote it in reduced form.**

8- Line 30: b-lactamase should be changed to β-lactamase.
Material and Methods

**Response –we thank you. Change has been made as β-lactamase as highlighted yellow**

9- The section “Study period and area” is partially missing.

**Response – yes, it was missing. We filed it appropriately here after. Thank you!**

10- It is not clear if “Sampling technique” is a section that includes the next ones, or if the content of this section is missing.

**Response – thank you again! we mean data extraction method from archived records of the laboratory**.
11- The authors should explain which antimicrobial susceptibility test and how they performed it.

**Response – thank you for the comment. The test protocol for the antimicrobial susceptibility test was Kirby-Bauer disc diffusion method.**

12- Line 12: The authors may scape the r from strains.

**Response- thanks you! Corrected as highlighted in the text as ‘strains’
13- The authors should explain what statistical test they have used.**

Descriptive ,crosstab, chi-square ,ratio, proportion,
Results
14- It would be interesting if the authors could provide data relating the resistance rates of the strains with the empirical treatment received by the patients.

**Response – thank you! , we have included that in figure 2.**
15- Figure 1: The authors should include the complete name of the labels.

**Response- thank you we wrote the complete name**
16- Line 9: The authors should explain the meaning of CSF.

**Response – thank you! we mean cerebrospinal fluid as highlighted in the text yellow.**
17- Line 11: Why the authors tested carbapenem susceptibility only in 37 strains, instead of in all the 102 samples?

**Response –thank you! There was a shortage of the carbapenem drug during testing.**
18- Line 11: The reference to Figure 2 should be in the previous sentence.

**Response – thank you! We have moved fig 2 to previous**
19- The percentage of ceftriaxone for 2018 in the text does not correspond with the 2018 percentage in the table.

**Response –thank you! We mean 98.6% and that has been corrected**
20- Figure 2: The authors should explain the meaning of EPHI.

**Response –thank you we mean Ethiopian public health Institute.**
21- Figures 3 and 4: The authors should include the label of the Y-axis.

**Response –thank you we included the label of Y-axis.**
22- Figures 3 and 4: I recommend the elimination of the grey background from both figures to facilitate the differentiation between the different line colours.

**Response –thank you we eliminated the background color.**
23- Figures 3 and 4: the meaning of the antibiotic acronyms should be explained.

**Response –thank you, we wrote in long form.**
Discussion
24- Line 10: What is the meaning of x^2?

**Response –thank you x^2^ we mean a symbol of chi-square**
25- The authors should revise the style and the content of the discussion, especially the last three paragraphs.

**Response –thank you so much we have corrected the last three paragraphs**

Reviewer #2: English needs improvement.

**Response –thank you we have attempted to revise the English grammar and spelling errors.**
Absolute numbers may not be mentioned when percentage is given.

**Response –thank you we opted to use percentage and edit again in the abstract and result section**

Introduction: at risk people may be described in a single sentence.

**Response:Thank you for the comments. Correction has been made per the comment given.**
Study period: the sentence seems incomplete.

**Response .thank you, it was missed we incorporated and fill the incomplete as highlighted yellow**
More than half of the samples were collected from two hospitals. How was the hospitals selected for this study?

**Response –thank you, the study included all health facilities referring specimens however specimens were no growth for *Acinetobacter* species during culturing in some facilities. They are high load government hospitals refer sample for microbiology culture and antimicrobial susceptibility testing. This study analyzed the isolates only i.e. we excluded the no growth specimens**
Figures should be in percentage.

**Response .Thank you we have made change to percentage**
The references are not uniformly written.

**Response –thank you we revised and have written it in Vancouver style.**
