# Supplementary material for: Multidrug resistance pattern of Acinetobacter species isolated from clinical specimens referred to the Ethiopian Public Health Institute: 2014 to 2018 trend anaylsis
Source: PLoS One. 2021 Apr 29;16(4):e0250896. doi: 10.1371/journal.pone.0250896 (PMC8084144; doi:10.1371/journal.pone.0250896)
Supplement: Attachment — Submitted filename: Response to Reviewers.docx [file pone.0250896.s003.docx]

Review Comments to the Author

Reviewer #1: The paper has been clearly improved, but I would recommend the authors to revise again due to some italics, antibiotic names as well as some spaces that are missing.

**Response – thank you! We have made changes in our revision some italics, antibiotic names as well as some spaces missed.**

Reviewer #3: the manuscript needs some attention on the following -
1. Introduction- please clearly define the multi-drug resistance of Acinetobacter species.
globally and regional data supporting the severity of multi-drug resistance of the organism (eg-mortality or
morbidity) would make the context stronger.

**Response – thank you for the comments to be added and we incorporate definition of MDR –*Acinetobacter* species, global and regional data supporting the severity of the disease in the introduction part.**
2. Methods- Inclusion criteria are not mentioned clearly.

**Response – thank you we have added the inclusion criteria of the study in method part**
3. Discussion- The result of empirical therapy is not discussed. please discuss with significance.

**Response – thank you for the comment regarding missed discussion of empirical therapy. We have added with significance in line number 205 on page 11**

4. Conclusion- Please rephrase the 1st line of conclusion.

**Response – we rephrase the 1st line of conclusion in line 243 on page 13 of the manuscript.**
